# Supplementary material for: Differential Alternative Splicing Genes in Response to Boron Deficiency in Brassica napus
Source: Genes (Basel). 2019 Mar 18;10(3):224. doi: 10.3390/genes10030224 (PMC6471828; doi:10.3390/genes10030224)
Supplement: Supplementary file 1 [file genes-10-00224-s001.zip › Supplementary materials-Jin Gu/Supplementary Figures-Jin Gu/Figure S1.docx]

| 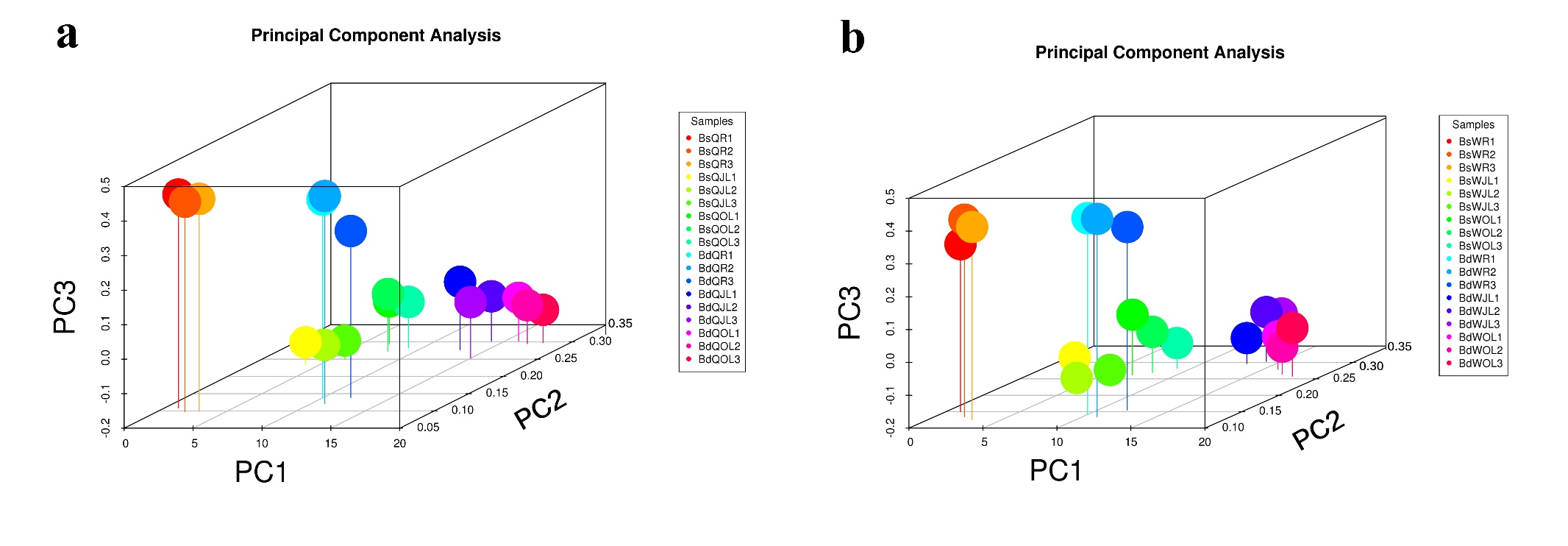 |
| --- |

Figure S1: Principal component analysis on all the RNA-Seq. data of the root, old leaves and juvenile leaves of Qingyou10 (a) and Westar10 (b) under B sufficient and deficient conditions. Bs, boron sufficient condition; Bd, boron deficient condition; QR, root of QY10; QOL, old leaves of QY10; QJL, juvenile leaves of QY10; WR, root of W10; WOL, old leaves of W10; WJL, juvenile leaves of W10. 1, 2 and 3 indicates replication 1, 2 and 3, respectively.
