# Supplementary material for: Differential Alternative Splicing Genes in Response to Boron Deficiency in Brassica napus
Source: Genes (Basel). 2019 Mar 18;10(3):224. doi: 10.3390/genes10030224 (PMC6471828; doi:10.3390/genes10030224)
Supplement: Supplementary file 1 [file genes-10-00224-s001.zip › Supplementary materials-Jin Gu/Supplementary Figures-Jin Gu/Figure S2.docx]

| 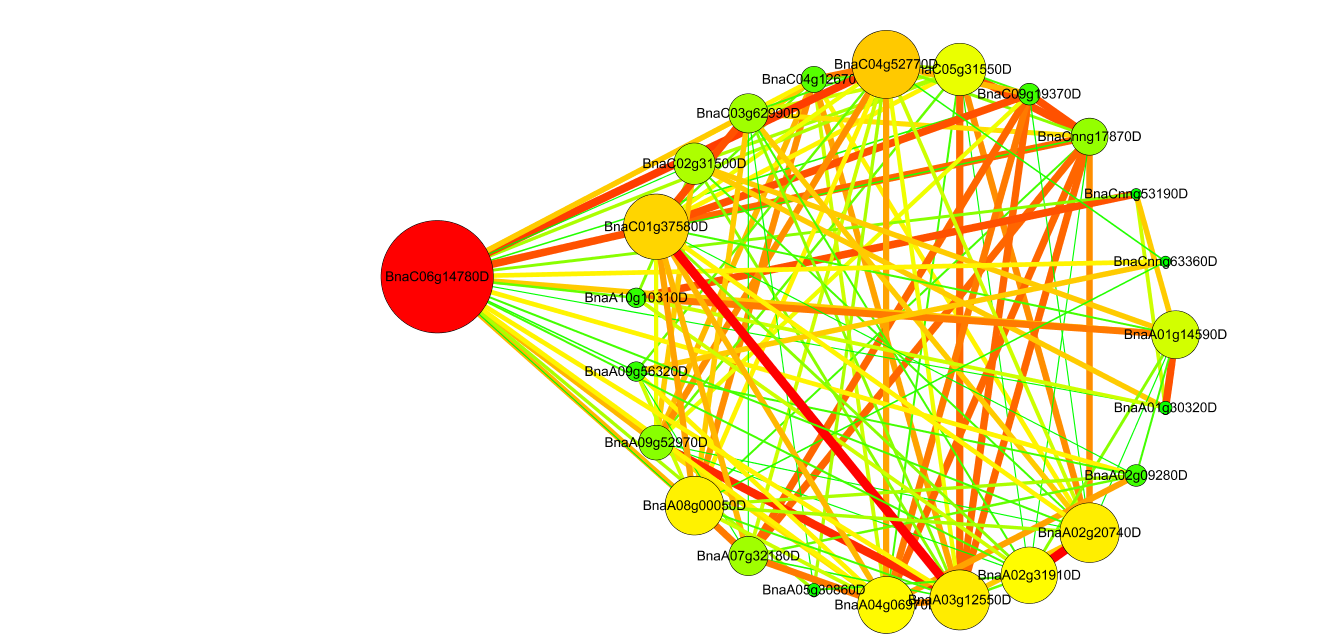 |
| --- |

Figure S2: The network of SR splicing factors and their target genes in Brassica napus at B deficient condition. Cytoscape software was used for visualizing the network (http://plantgrn.noble.org/DeGNServer/Analysis.jsp).
