# Supplementary material for: Differential Alternative Splicing Genes in Response to Boron Deficiency in Brassica napus
Source: Genes (Basel). 2019 Mar 18;10(3):224. doi: 10.3390/genes10030224 (PMC6471828; doi:10.3390/genes10030224)
Supplement: Supplementary file 1 [file genes-10-00224-s001.zip › Supplementary materials-Jin Gu/Supplementary Tables-Jin Gu/Table S5.doc]

**Differential alternative splicing genes in response to boron deficiency in *Brassica napus***

Jin Gu, Wei Li, Sheliang Wang, Xiaoyan Zhang, Anne Coules, Guangda Ding, Fangsen Xu, Jian Ren, Chungui Lu, Lei Shi

**Table S5 Annotation of the genes in transcriptional and posttranscriptional regulation in the root, juvenile leaves and old leaves of *Brassica napus* cultivars Qingyou10 and Westar10 at boron deficient conditions.**

| Tissues | | Gene ID | Gene Annoation | RPKM | | DAS Type-Regulation | | |
| --- | --- | --- | --- | --- | --- | --- | --- | --- |
|  |  |  |  | B Deficient/B Sufficient | |  |  |  |
| QR | BnaA01g30320D | | phosphoglycerate kinase 1 (PGK1) | | - | | DA3SS |  |
|  | BnaC09g01100D | | GDP-fucose protein O-fucosyltransferase | | 5.19 | | - |  |
|  | BnaA01g30340D | | SANT/Myb domain | | 3.48 | | DA3SS |  |
|  | BnaC08g37640D | | CCAAT-binding transcription factor | | 4.47 | | DA3SS |  |
|  | BnaA03g07610D | | Translation elongation factor EF1B | | 3.75 | | DA3SS |  |
|  | BnaA04g02970D | | Calcium-dependent phosphotriesterase superfamily protein | | - | | DIR |  |
|  | BnaA07g33860D | | Sulphate anion transporter | | 0.09 | | DIR |  |
|  | BnaA02g03490D | | Oxidoreductase-like, N-terminal | | 0.41 | | - |  |
|  | BnaA09g52970D | | Expansin | | 0.03 | | DIR |  |
|  | BnaA05g06290D | | Calmodulin-binding domain, plant | | 8.12 | | - |  |
|  | BnaC03g47960D | | Oxoglutarate/iron-dependent dioxygenase | | 0.51 | | DA3SS |  |
|  | BnaC06g27340D | | Thioredoxin-like fold | | 6.42 | | - |  |
|  | BnaC08g35100D | | Glutathione S-transferase, C-terminal-like | | 2.96 | | - |  |
|  | BnaC04g06320D | | Amino acid transporter, transmembrane | | 0.41 | | DRI |  |
|  | BnaC04g56100D | | Glycosyl hydrolases 36 | | 5.50 | | - |  |
|  | BnaA06g07200D | | aconitase 3 (ACO3) | | - | | DIR |  |
|  | BnaA04g27220D | | Galactosyl transferase | | 4.66 | | - |  |
|  | BnaA06g17710D | | alpha-glucan phosphorylase 2 (PHS2) | | - | | DIR |  |
|  | BnaA05g21970D | | Glycosyltransferase AER61, uncharacterised | | 0.04 | | - |  |
|  | BnaA01g10000D | | Pectinacetylesterase | | 3.04 | | - |  |
|  | BnaA06g10710D | | magnesium transporter 2 (MGT2) | | - | | DIR |  |
|  | BnaA01g08470D | | Aminotransferase, class I/classII | | 0.14 | | - |  |
|  | BnaA04g01170D | | Auxin-induced protein, ARG7 | | 2.10 | | - |  |
|  | BnaA03g42620D | | Sugar/inositol transporter | | 4.35 | | - |  |
|  | BnaA06g30540D | | NADH-ubiquinone oxidoreductase B8 subunit, putative | | - | | DES |  |
|  | BnaA07g16850D | | DNA-binding WRKY | | 10.51 | | - |  |
|  | BnaC03g74670D | | Transferase | | 5.14 | | - |  |
|  | BnaC04g35920D | | Protein kinase superfamily protein | | - | | DRI |  |
|  | BnaC08g00880D | | Pectate lyase/Amb allergen | | 10.71 | | - |  |
|  | BnaC05g03300D | | Plant peroxidase | | 3.68 | | - |  |
|  | BnaC07g49360D | | Protein kinase domain | | 7.14 | | - |  |
|  | BnaC05g08610D | | sugar transporter 1 (STP1) | | - | | DA3SS |  |
|  | BnaA02g22030D | | Major intrinsic protein | | 3.42 | | - |  |
|  | BnaC01g06090D | | Cellulose synthase | | 0.43 | | - |  |
|  | BnaC05g18490D | | Phosphoglucomutase/phosphomannomutase family protein | | - | | DIR |  |
|  | BnaC03g25710D | | Arabinogalactan peptide, AGP | | 0.41 | | - |  |
|  | BnaC07g16140D | | Xylan biosynthesis protein IRX15/IRX15L | | 0.17 | | - |  |
|  | BnaAnng10900D | | Cation-transporting P-type ATPase | | 0.01 | | - |  |
|  | BnaC08g42250D | | zinc transporter 4 precursor (ZIP4) | | - | | DA3SS |  |
|  | BnaC08g29490D | | citrate synthase 2 (CSY2) | | - | | DIR |  |
| QJL | BnaA01g02090D | | calmodulin-domain protein kinase 5 (CPK5) | | - | | DRI |  |
|  | BnaA03g07610D | | Translation elongation factor EF1B | | - | | DA3SS |  |
|  | BnaA10g25990D | | C2 calcium-dependent membrane targeting | | 11.84 | | - |  |
|  | BnaA01g29470D | | Pectinesterase, catalytic | | 9.55 | | - |  |
|  | BnaA07g16660D | | Fructose-1,6-bisphosphatase class 1 | | 0.19 | | - |  |
|  | BnaA07g35330D | | Major intrinsic protein | | 8.32 | | - |  |
|  | BnaA07g17230D | | Glycoside hydrolase, family 17 | | 3.68 | | - |  |
|  | BnaA03g10160D | | Protein kinase domain | | 2.10 | | - |  |
|  | BnaAnng08760D | | Nucleotidyl transferase | | 0.18 | | DA3SS |  |
|  | BnaCnng52390D | | Pollen Ole e 1 allergen/extensin | | 0.27 | | DES |  |
|  | BnaA01g20660D | | Plant peroxidase | | 2.30 | | - |  |
|  | BnaC01g01940D | | SANT/Myb domain | | 6.31 | | - |  |
|  | BnaC06g33980D | | CCAAT-binding transcription factor, subunit B | | 6.10 | | - |  |
|  | BnaC02g16920D | | Protein kinase superfamily protein | | - | | DA3SS |  |
|  | BnaA03g07660D | | Sulphate anion transporter | | 4.87 | | - |  |
|  | BnaC04g05770D | | Expansin | | 4.25 | | - |  |
|  | BnaC04g43040D | | cellulose synthase-like B3 (CSLB03) | | - | | DIR |  |
|  | BnaC05g08610D | | sugar transporter 1 (STP1) | | - | | DA3SS |  |
|  | BnaA04g17420D | | DNA-binding WRKY | | 9.44 | | - |  |
|  | BnaC06g42250D | | Glycerol-3-phosphate dehydrogenase, NAD-dependent | | 0.28 | | - |  |
|  | BnaC05g48310D | | 6-phosphogluconate dehydrogenase, C-terminal | | 3.08 | | - |  |
|  | BnaA08g27070D | | Amino acid transporter, transmembrane | | 5.79 | | - |  |
|  | BnaC04g26530D | | Fructose-1,6-bisphosphatase class 1 | | 0.23 | | DES |  |
|  | BnaC05g06170D | | Magnesium chelatase, ChlI subunit | | 0.30 | | DA3SS |  |
|  | BnaAnng37730D | | glutathione S-transferase phi 8 (GSTF8) | | - | | DES |  |
|  | BnaC09g01100D | | GDP-fucose protein O-fucosyltransferase | | 9.10 | | - |  |
| QOL | BnaA08g11360D | | C2 calcium-dependent membrane targeting | | 0.16 | | DRI |  |
|  | BnaA09g42040D | | Cation-transporting P-type ATPase | | 17.69 | | DA3SS |  |
|  | BnaA01g02090D | | calmodulin-domain protein kinase 5 (CPK5) | | - | | DRI |  |
|  | BnaA02g29830D | | Glycosyl transferase, family 35 | | - | | DRI |  |
|  | BnaA03g07610D | | Translation elongation factor EF1B | | - | | DA3SS |  |
|  | BnaC05g49110D | | Zinc/iron permease | | 6.94 | | DA3SS |  |
|  | BnaAnng37730D | | Glutathione S-transferase, N-terminal | | 2.42 | | DES |  |
|  | BnaA03g21140D | | Pectinesterase, catalytic | | 4.71 | | - |  |
|  | BnaC04g02330D | | protein phosphatase 2A-3 (PP2A-3) | | - | | DES |  |
|  | BnaA03g24370D | | Major intrinsic protein | | 12.92 | | - |  |
|  | BnaC04g33570D | | Fructose-bisphosphate aldolase, class-I | | 2.20 | | - |  |
|  | BnaC05g08610D | | sugar transporter 1 (STP1) | | - | | DA3SS |  |
|  | BnaC01g13370D | | Plant peroxidase | | 3.92 | | - |  |
|  | BnaA09g44370D | | SANT/Myb domain | | 7.88 | | - |  |
|  | BnaA04g22910D | | Expansin | | 4.18 | | - |  |
|  | BnaA05g32660D | | Transferase | | 6.11 | | - |  |
|  | BnaC03g49470D | | DNA-binding WRKY | | 3.28 | | - |  |
|  | BnaA06g14680D | | Sugar/inositol transporter | | 0.21 | | - |  |
|  | BnaC02g15560D | | Aminotransferase, class I/classII | | 3.47 | | - |  |
|  | BnaC04g43040D | | Cellulose synthase | | 2.30 | | - |  |
|  | BnaC06g35300D | | Galactosyl transferase | | 0.31 | | - |  |
| WR | BnaA03g07610D | | Translation elongation factor EF1B | | - | | DA3SS |  |
|  | BnaA06g07000D | | Protein kinase domain | | 5.89 | | DRI |  |
|  | BnaA04g02970D | | Calcium-dependent phosphotriesterase superfamily protein | | - | | DRI |  |
|  | BnaC04g30460D | | C2 calcium-dependent membrane targeting | | 3.45 | | - |  |
|  | BnaA07g33860D | | Sulphate anion transporter | | 3.30 | | DRI |  |
|  | BnaAnng07200D | | Nucleic acid-binding, OB-fold | | 0.28 | | DA3SS |  |
|  | BnaA01g07610D | | Phosphofructokinase domain | | 4.58 | | - |  |
|  | BnaA02g10290D | | Pectinesterase, catalytic | | 0.34 | | - |  |
|  | BnaC03g04980D | | Glycoside hydrolase, family 31 | | 3.56 | | - |  |
|  | BnaC03g39560D | | Major intrinsic protein | | 0.01 | | - |  |
|  | BnaC02g02590D | | SANT/Myb domain | | 6.31 | | - |  |
|  | BnaA06g08040D | | Expansin | | 0.25 | | - |  |
|  | BnaA09g27140D | | Transferase | | 0.42 | | - |  |
|  | BnaA06g28860D | | Sugar/inositol transporter | | 2.89 | | - |  |
|  | BnaC04g21220D | | Glutathione S-transferase, N-terminal | | 0.07 | | - |  |
|  | BnaC01g15640D | | DNA-binding WRKY | | 6.54 | | - |  |
| WJL | BnaC06g09450D | | Mannose-binding lectin | | N/A | | DES |  |
|  | BnaA03g55140D | | phosphoglucomutase (PGM) | | - | | DRI |  |
|  | BnaC03g32010D | | C2 calcium-dependent membrane targeting | | 6.39 | | - |  |
|  | BnaA10g06710D | | Pectinesterase, catalytic | | 4.15 | | - |  |
|  | BnaA09g36820D | | calcium-dependent protein kinase 32 (CPK32) | | - | | DRI |  |
|  | BnaC06g42620D | | Fructose-1,6-bisphosphatase class 1 | | 0.34 | | - |  |
|  | BnaC04g24330D | | Glycoside hydrolase, family 17 | | 3.56 | | - |  |
|  | BnaA08g12770D | | Protein kinase domain | | 4.25 | | - |  |
|  | BnaC05g00440D | | plasma membrane intrinsic protein 1 | | - | | DRI |  |
|  | BnaA03g40690D | | SANT/Myb domain | | 0.24 | | - |  |
|  | BnaC03g22200D | | Expansin | | 3.57 | | - |  |
|  | BnaC01g41490D | | Transferase | | 0.18 | | - |  |
|  | BnaC07g38550D | | DNA-binding WRKY | | 4.31 | | - |  |
|  | BnaC08g42910D | | Sugar/inositol transporter | | 5.67 | | - |  |
|  | BnaA09g19060D | | Glutathione S-transferase, N-terminal | | 4.38 | | - |  |
|  | BnaC04g06320D | | Amino acid transporter, transmembrane | | 7.38 | | - |  |
| WOL | BnaA06g17710D | | Glycosyl transferase, family 35 | | 4.24 | | DA3SS |  |
|  | BnaA01g02090D | | calmodulin-domain protein kinase 5 (CPK5) | | - | | DRI |  |
|  | BnaC06g24480D | | C2 calcium-dependent membrane targeting | | 0.21 | | - |  |
|  | BnaA04g24790D | | Pectinesterase, catalytic | | 4.61 | | - |  |
|  | BnaC02g29210D | | Major intrinsic protein | | 4.90 | | - |  |
|  | BnaC08g28170D | | Glycoside hydrolase, family 17 | | 5.29 | | - |  |
|  | BnaAnng37730D | | glutathione S-transferase phi 8 (GSTF8) | | - | | DES |  |
|  | BnaC05g18490D | | Phosphoglucomutase/phosphomannomutase family protein | | - | | DRI |  |
|  | BnaA08g17100D | | Protein kinase domain | | 0.27 | | - |  |
|  | BnaA01g11800D | | Plant peroxidase | | 7.75 | | - |  |
|  | BnaA07g11710D | | SANT/Myb domain | | 0.22 | | - |  |
|  | BnaA03g35980D | | CCAAT-binding transcription factor, subunit B | | 0.48 | | - |  |
|  | BnaA04g22910D | | Expansin | | 10.76 | | - |  |
|  | BnaC09g48280D | | Transferase | | 0.10 | | - |  |
|  | BnaA09g00350D | | DNA-binding WRKY | | 3.91 | | - |  |
|  | BnaA09g48650D | | Sugar/inositol transporter | | 0.06 | | - |  |
|  | BnaC03g03750D | | Amino acid transporter, transmembrane | | 0.10 | | - |  |

Note: DAS, differential alternative splicing; DE, differential expressed; Bs, B sufficient conditions; Bd, B deficient conditions; QR, root of QY10; QOL, old leaves of QY10; QJL, juvenile leaves of QY10; WR, root of W10; WOL, old leaves of W10; WJL, juvenile leaves of W10; N/A, the genes with up-regulated expression at B deficient condition and no expression at B sufficient conditions; DA3SS, differential alternative 3’ splicing site; DA5SS, differential alternative 5’ splicing site; DRI, differential retain intron; DES, differential exon skipping; -, No DAS or DE of the gene.
