# Supplementary material for: Differential Alternative Splicing Genes in Response to Boron Deficiency in Brassica napus
Source: Genes (Basel). 2019 Mar 18;10(3):224. doi: 10.3390/genes10030224 (PMC6471828; doi:10.3390/genes10030224)
Supplement: Supplementary file 1 [file genes-10-00224-s001.zip › Supplementary materials-Jin Gu/Supplementary Tables-Jin Gu/Table S6.doc]

**Differential alternative splicing genes in response to boron deficiency in *Brassica napus***

Jin Gu, Wei Li, Sheliang Wang, Xiaoyan Zhang, Anne Coules, Guangda Ding, Fangsen Xu, Jian Ren, Chungui Lu, Lei Shi

**Table S6 The types of differential alternative splicing of SR splicing factor in *Brassica napus* cultivars Qingyou10 and Westar10 at boron deficient conditions**

| Gene ID | Gene NO. | Gene Name | Types of DAS | | | | | |
| --- | --- | --- | --- | --- | --- | --- | --- | --- |
|  |  |  | BsQR vs BdQR | BsQJL vs BdQJL | BsQOL vs BdQOL | BsWR vs BdWR | BsWJL vs BdWJL | BsWOL vs BdWOL |
| BnaC05g06630D | 2 | AT-SR30 | - | - | - | - | - | - |
| BnaA06g37780D |  |  | - | - | - | - | - | - |
| BnaA09g00790D | 2 | AT-SR34 | - | - | - | - | - | - |
| BnaA02g20550D |  |  | - | - | - | - | - | - |
| BnaC08g21130D | 2 | AT-SR34A | - | - | - | - | - | - |
| BnaA06g15930D |  |  | - | - | - | - | ES | - |
| BnaA02g20550D | 2 | AT-SR34B | - | - | - | - | - | - |
| BnaA09g00790D |  |  | - | - | - | - | - | - |
| BnaC05g19020D | 1 | AT-RSZ21 | - | - | - | - | - | - |
| BnaC07g43350D | 1 | AT-RSZ22 | - | - | - | - | - | - |
| BnaAnng28560D | 2 | AT-RSZ22A | - | - | - | - | - | - |
| BnaC08g33900D |  |  | - | - | - | - | - | - |
| BnaC09g39040D | 1 | AT-SCL28 | - | - | - | - | - | - |
| BnaC04g25450D | 2 | AT-SCL30 | - | - | - | - | - | - |
| BnaA04g03560D |  |  | - | - | - | - | - | - |
| BnaC01g37560D | 2 | AT-SCL30A | - | - | - | - | - | - |
| BnaCnng00990D |  |  | - | - | - | - | - | - |
| BnaA05g13830D | 2 | AT-SCL33 | - | - | - | - | - | - |
| BnaC06g10860D |  |  | - | - | - | - | - | - |
| BnaC08g24530D | 4 | AT-RS2Z | - | - | - | - | - | - |
| BnaA09g33780D |  |  | - | - | - | - | - | - |
| BnaA07g37700D |  |  | - | - | - | - | - | - |
| BnaC06g14780D |  |  | A3SS | A3SS | A3SS | - | - | - |
| BnaC03g20680D | 2 | AT-RS2Z33 | - | - | - | - | - | - |
| BnaA07g37700D |  |  | - | - | - | - | - | - |
| BnaC04g00810D | 2 | AT-RS31A | - | - | - | - | - | - |
| BnaA05g01240D |  |  | - | - | - | - | - | - |
| BnaA09g55560D | 1 | AT-RS31 | - | - | - | - | - | - |
| BnaC08g47240D | 4 | AT-RS40 | - | - | - | - | - | - |
| BnaA08g30960D |  |  | - | - | - | - | - | - |
| BnaA01g14750D |  |  | A5SS | - | - | - | - | - |
| BnaC01g41640D |  |  | ES | - | ES | - | - | - |
| BnaA03g12870D | 1 | AT-RS41 | - | - | - | - | - | - |
| BnaA06g23350D | 1 | AT-SC35 | - | - | - | - | - | - |

Note：DAS, differential alternative splicing; Bs, B sufficient conditions; Bd, B deficient conditions; QR, root of QY10; QOL, old leaves of QY10; QJL, juvenile leaves of QY10; WR, root of W10; WOL, old leaves of W10; WJL, juvenile leaves of W10; A3SS, alternative 3’ splicing site; A5SS, alternative 5’ splicing site; ES, exon skipping; “-”, non-DAS
